# Targeting of phosphatidylserine by monoclonal antibodies reverses an immunosuppressive checkpoint inducing innate and specific anti-tumor responses

**DOI:** 10.1186/2051-1426-1-S1-P151

**Published:** 2013-11-07

**Authors:** Jian Gong, Rich Archer, Van Nguyen, Jeff Hutchins, Bruce Freimark

**Affiliations:** 1 Preclinical Research, Peregrine Pharmaceuticals, Inc, Tustin, CA, USA

## 

Phosphatidylserine (PS) is a phospholipid normally residing in the inner leaflet of the plasma membrane and becomes exposed on tumor vascular endothelial cells (ECs) and tumor cells, and exposure is enhanced in response to chemotherapy, irradiation and oxidative stresses in the tumor microenvironment. PS exposure in tumors promotes an immunosuppressive microenvironment which includes the recruitment of myeloid derived suppressor cells (MDSCs) and M2-like macrophages as well as the production of anti-inflammatory cytokines. Binding of PS targeting antibodies on tumor endothelial cells, tumors and their secreted microparticles triggers a Fc-FcR mediated pro-inflammatory cellular and cytokine response that reverses this immunosuppressive, PS meditated checkpoint thereby enhancing anti-tumor immunity. A chimeric anti-PS antibody, bavituximab, is being used in combination with chemotherapy to treat patients with solid tumors in multiple late-stage clinical trials. Using syngeneic tumors and human tumor xenografts in mice, we demonstrate PS targeting antibodies specifically localize to PS exposed on membranes of tumor blood vessels, tumors, tumor-infiltrating inflammatory cells and microparticles. Analysis of blood, spleens and tumor tissue demonstrates that PS targeting antibodies are capable of suppressing tumor growth in multiple tumor types by several mechanisms including destruction of tumor blood vessels by ADCC mechanisms, blockage of the PS-mediated immunosuppressive checkpoint, and reactivation of M1 macrophages, dendritic cell maturation and T-cell cellular anti-tumor responses. The combination of these mechanisms promotes strong localized anti-tumor responses without the side-effects of systemic immune activation.

